# Announcement on Use of AI

**DOI:** 10.14252/foodsafetyfscj.D-25-00010

**Published:** 2025-03-21

## Abstract

In recent years, as significant progress has been made in artificial intelligence (AI) technology, various peer-reviewed journals have been extending the policies on use of AI. Considering this situation, ***Food Safety*** felt the need to address concerns about the use of the technologies such as Large Language Models (LLMs), chatbots, or image creators in the writing and reviewing of manuscripts. ***Food Safety***, thus, has compiled a tentative and preliminary guideline for responsible use of AI tools based on the ICMJE Recommendations. Authors and reviewers are expected to refer to this guideline during the manuscript preparation and peer-review process. Guide for Authors for ***Food Safety*** will be updated in response to the comments from authors and reviewers, as well as updates on the ICMJE Recommendations and policies of other journals. Please feel free to contact Editorial Office of ***Food Safety*** if you have any comments or questions about the guideline.

## 1. For Authors

In consonance with the ICMJE Recommendations^1^^)^, ***Food Safety*** does not allow AI to be listed as an author or co-author. AI cannot be responsible for the accuracy, integrity, and originality of the work, thus they do not meet the ICMJE’s criteria for authorship^1^^)^. Authors who use AI should describe, in both the cover letter and the submitted work in the Materials and Methods section, Acknowledgment or appropriate section if applicable, how they used it. Nonetheless the submission of images created by AI is discouraged. Authors are fully responsible for any material submitted whether it is prepared with the use of AI tools or not. They should be able to assert that there is no plagiarism in their manuscript including text, figures and images.

## 2. For Peer-Reviewers

Reviewers must maintain the confidentiality of the manuscript as outlined in ICMJE Recommendations^2^^)^.

This responsibility prohibits reviewers from uploading manuscript to AI where confidentiality cannot be assured. For other potential use of AI tools, reviewers are requested to contact the Editorial Office.
